# What the fruit fly can tell us about autosomal recessive primary microcephaly

**DOI:** 10.1080/19336934.2025.2572866

**Published:** 2025-10-11

**Authors:** Shalini Chakraborty, Steven Florez, Todd Schoborg

**Affiliations:** Department of Molecular Biology, University of Wyoming, Laramie, WY USA

**Keywords:** *Drosophila*, neurogenesis, neuroblasts, neural stem cells, microcephaly, MCPH, abnormal spindle, ASPM, microcephalin, wdr62, ankle2

## Abstract

Three decades of research aimed at understanding the basis for autosomal recessive primary microcephaly (MCPH), a human clinical disorder defined by a significant reduction in head and brain size, has uncovered a suite of ~30 genes that participate in this process. Work in both vertebrate and invertebrate model systems have been instrumental in attempting to link MCPH gene function to the brain growth phenotype. However, we still lack definitive evidence as to what these functions are for many of these genes. In this review, we summarize recent work in *Drosophila* aimed at overcoming these limitations in our knowledge of MCPH gene function that may be applicable to humans. We discuss the clinical features of MCPH, parallels between human and *Drosophila* neurogenesis modes with a particular focus on the fly optic lobe, and highlight four of the most well-studied *Drosophila* MCPH orthologs: *abnormal spindle (asp)/MCPH5, Microcephalin/MCPH1, WD Repeat-Containing Protein 62 (Wdr62)/MCPH2*, and *Ankryin Repeat-and LEM Domain- Containing Protein 2 (ANKLE2)*/*MCPH16*. We focus on the multifunctional roles for these proteins that may underlie the microcephaly phenotype and advocate for the use of flies as a relevant model for human MCPH.

## Introduction

It has been nearly 30 years since the first genetic locus responsible for autosomal recessive primary microcephaly (MCPH) was identified [1]. Since then, 30 genes have been given an official MCPH designation in OMIM [2]. While we now have a greater understanding of the genes involved, their phenotypic relationship to the small brains observed in human patients is less clear. This is primarily due to two factors: the diversity of cellular roles that these genes are known to participate in and the complex nature of the brain’s developmental process, which must be precisely coordinated in time and space to ensure that efficient neurogenesis is translated into a properly sized brain.

A multitude of biological factors contribute to this complexity of brain development, including coordination of cell cycle and developmental timing, mitotic spindle orientation, transcriptional and epigenetic gene regulation, cell signalling pathways and neuronal migration (reviewed in [3]). Additionally, the heterogeneous nature of clinical microcephaly makes it extremely difficult to delineate the potential causes of this disorder, given that the same small brain size feature (a clinical hallmark of microcephaly) might arise from disruptions in entirely different biological processes, making it difficult to pinpoint a single molecular signature for this disorder.

However, clues from evolutionary cell biology may provide a suitable framework for understanding how cellular behaviour influences brain size. In terms of the cellular ‘scaling factors’ that correlate with brain size, primate brain size is largely driven by total cell number, as opposed to rodent brains, which tend to increase cell size in order to increase brain volume [4–7]. Therefore, a simplified and cohesive view of the MCPH gene-phenotype relationship is that any disruption to the production of brain cells in a timely manner leads to a small brain.

However, the devil lies in the mechanistic details. The microcephaly field was driven largely by correlative studies during the first two decades of its existence, relying on the most well-known role (at the time) of the MCPH gene in question to formulate a hypothesis for how it’s loss caused a small brain [8]. While these hypotheses made sense intuitively and provided the framework that later studies would eventually put to the test, the limitations of such an approach has become clear in recent years as a number of newer studies have failed to definitively link these well-known MCPH gene roles to the microcephaly phenotype [9–12].

These studies also revealed that protein multifunctionality (e.g. moonlighting) may be a significant contributor to the microcephaly phenotype. These secondary roles allow more complex biological functions from a smaller suite of genes and are becoming increasingly more relevant in genetic causes of human disease, especially those for which a single gene mutation is thought to be responsible for the disease phenotype [13,14]. This highlights a critical limitation of relying on correlation to explain the aetiology of MCPH – without complete knowledge of a protein’s entire set of functions, we cannot understand the fundamental biology behind the disorder and therefore lack any viable approach towards therapeutic intervention.

However, characterizing multifunctional protein roles is challenging. Phenotypes can be subtle and/or difficult to definitely link to the mutant phenotype in question, may be cell or tissue-specific, or only occur with specific binding partners. We also lack suitable computational tools to predict protein secondary functions [15]. These challenges therefore necessitate the use of a suitable model organism that possesses the necessary genetic tools to properly identify and detail the mechanism by which these secondary functions impact tissue development, particularly for the brain. But therein lies a bigger problem when trying to understand these roles in the context of human brain growth control – can the superior genetic tools of model organisms really elucidate these mechanisms when brain size is a defining feature of being human [16]?

In this review, we advocate for the use of the fruit fly (*Drosophila melanogaster*) as a suitable model system for human MCPH. We describe the fly optic lobe and its development to highlight parallels to mammalian brain development, including human-specific features such as the relationship between brain cell number and tissue volume. We then summarize recent studies from four of the most well-studied MCPH orthologs in flies (MCPH1-*Microcephalin*, MCPH5-*Abnormal Spindle (Asp)*, MCPH2*-WD Repeat-Containing Protein 62 (WDR62)*, and MCPH16-*ANKLE2*), highlighting multifunctional roles for these proteins that may be the key for understanding the molecular basis of MCPH.

## Microcephaly: a clinical definition for human patients

Microcephaly is a rare neurodevelopmental disorder which is primarily characterized by a reduction in brain volume, particularly the cerebral cortex, various forms of intellectual disabilities, and life span reduction. The clinical definition of microcephaly includes a reduction in occipital frontal head circumference (OFC) that is greater than two standard deviations (SD) below the mean for an individual’s age, sex and ethnicity [17–20]. An OFC greater than 3 SDs below the mean signifies a ‘severe’ form of microcephaly. Microcephaly can be divided into two major categories: congenital/primary (if it appears at birth) and secondary (postnatally). Congenital microcephaly is primarily a neurodevelopmental defect. However, secondary microcephaly is often considered to be neurodegenerative and progressive in nature [21]. Both primary and secondary microcephaly can be genetic or acquired [22].

A wide variety of environmental factors can lead to primary microcephaly. Exposure of the foetus to infections during pregnancy, particularly from agents of the ToRCH complex (Toxoplasma (To), Rubella Virus (R), Cytomegalovirus (C) and Herpes Simplex Viruses (H)), and more recently, Zika virus, can disrupt brain development and cause microcephaly [23,24]. Foetal exposure to toxins like alcohol, certain drugs, heavy metals like mercury, radiation and chemicals in tobacco smoke have also been documented to cause microcephaly [25–27]. Primary microcephaly can further be subdivided into three categories: 1) isolated microcephaly, referring to only small brain size and no further clinical abnormalities like developmental and intellectual disabilities; 2) non-syndromic microcephaly, referring to a condition with neurological defects but without cerebral or extra-cerebral abnormalities; 3) syndromic microcephaly with cerebral malformations and/or extra-cerebral anomalies like facial dysmorphism, eye, ear, heart disorders, etc. [28].

Primary microcephaly is generally considered an autosomal recessive trait, which suggests the affected individual will only develop the condition if he/she inherits a copy of the mutated gene from each parent [29]. However, a small portion of MCPH genes have also been linked to autosomal dominant conditions, in which case only one copy of the gene is sufficient to cause the disease (e.g. *LMNB2, WDFY3*). Nonetheless, autosomal recessive primary microcephaly (MCPH) is largely associated with single gene mutations that behave recessively, often within consanguineous relationships, resulting in isolated microcephaly within a lineage [25].

## Fruit flies, a powerful genetic model to study neurodevelopmental disorders

The human brain is a complex organ that functions to coordinate body locomotion and movement, interpret various senses, and facilitate normal behaviour and cognitive responses. Abnormalities in proper brain growth and development form the basis of various neurodevelopmental disorders (NDDs) like microcephaly. An approach towards understanding these incurable disorders is to determine molecular and cellular mechanisms of NDDs in less complex organisms that have sophisticated nervous systems.

Fruit flies, a highly genetically tractable animal model, have been employed over the years to study human disease, including NDDs. Fruit flies have numerous experimental advantages to conduct NDD research, such as a well-characterized genome amenable to genetic manipulation, a short 10-day life cycle, production of progeny in large numbers, and most importantly, a wide variety of powerful genetic tools to elucidate molecular mechanisms behind human diseases [30].

In addition to these technical advantages, there are other reasons why flies are powerful for studying NDDs. First, *Drosophila* shares many conserved genetic pathways that are involved in neurodevelopment with humans [31,32]. About 75% of the genes known to be involved in human NDDs have orthologs in *Drosophila* [33,34]. Secondly, although the fly’s neuroanatomy is comparatively simpler than that of humans, its central nervous system (CNS) still shares many key characteristics with the human brain. For example, flies have well-defined neural circuits to understand behaviour, and complete connectomes providing synapse-level resolution are available for both the larval and adult brain [35,36]. Additionally, they exhibit complex behaviours such as learning, memory, and locomotion, which can be used to model neurodevelopmental disorders that impact cognitive behaviour [37–40]. Combined with the sophisticated genetic tools available, flies can serve as an attractive model to uncover the mechanistic basis of these processes that are relevant to humans and NDDs.

**Figure 1.**
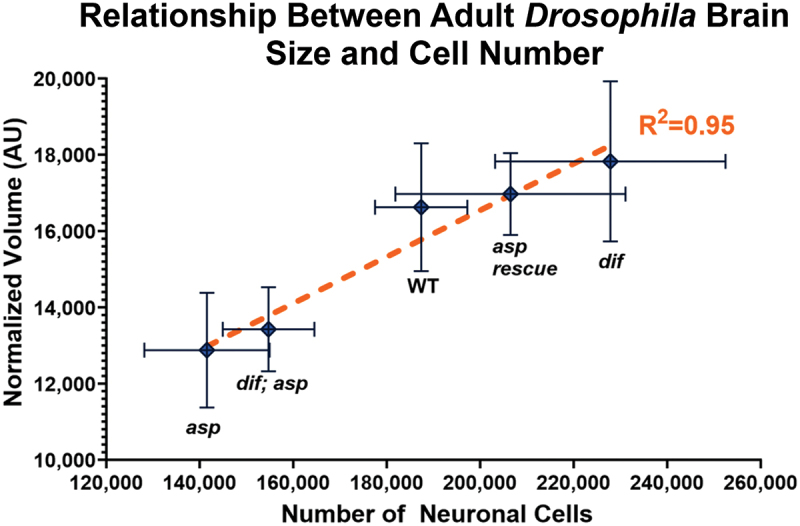
*Drosophila melanogaster* adult brain size strongly correlates with total neuronal cell number. Graph shows the relationship between adult brain size as measured by microcomputed tomography and total neuronal cell number as measured by flow cytometry for the indicated genotypes: *asp (abnormal spindle* mutant), *dif; asp* (*dorsal-related immunity factor; abnormal spindle* double mutants), yellow-white (WT), *asp rescue* (*abnormal spindle* mutants expressing a rescue transgene), *dif* (*dorsal-related immunity factor* single mutant) [41]. For each genotype, *n* > 10 single brains were analysed. Simple linear regression (R^2^) was performed using PRISM software.

This is especially true for NDDs related to brain growth control, such as MCPH. Unlike rodents, which utilize increases in cell size to produce larger brains, fly brains show a strong correlation between total brain cell number and total volume (Figure 1), much like primates [4–7]. This may explain why non-primate vertebrate models, such as mice and zebrafish, show relatively subtle brain size decreases when MCPH genes such as MCPH5 (*Abnormal Spindle-Like, Microcephaly Associated (ASPM)* and MCPH2 (*WDR62)* are mutated [10,42–44]. Flies, on the other hand, show a strong reduction in brain size ( > 30% for the adult optic lobes) that correlates with a > 30% decrease in total brain cell number when the MCPH5 ortholog *Abnormal Spindle (asp)* is mutated [41,45]. Such strong size phenotypes therefore allow even subtle perturbations to be investigated. Furthermore, genetic rescue experiments in flies using human transgenes of MCPH5 (*ASPM*) and MCPH16 (*ANKLE2*) can compensate for fly-specific mutations and largely restore brain size, providing strong evidence that the mechanisms promoting proper brain growth are conserved across > 700 million years of evolution [46–48]. Finally, flies have proven to be a useful system for evaluating the genetic contributions to other human disorders that include microcephaly as a clinical feature, such as the Microcephalic Primordial Dwarfism (PD) disorders (Seckel syndrome, Meier-Gorlin syndrome, and microcephalic osteodysplastic PD types I and II (MOPD I and II)) [49–53].

## Mechanisms of mammalian cerebral cortex development

**Figure 2.**
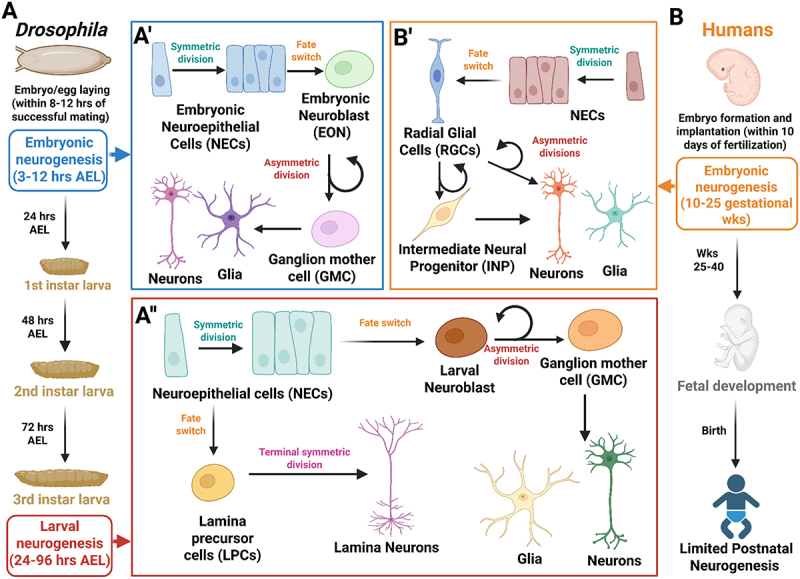
Comparative timeline and neurogenesis mechanisms in flies and humans. Cartoon highlighting the general features of neurogenesis. (A) in flies, two neurogenesis periods exist. Embryonic neurogenesis occurs from 3–12 hours after egg laying (AEL) followed by a short quiescent period before larval neurogenesis begins during the 1^st^ and 2^nd^ larval instars. Larval neurogenesis peaks during the 3^rd^ instar stage (~72 hrs AEL) with most neurons and glia being generated during the late third instar period (72–96 hrs AEL). (A’) during embryonic neurogenesis, symmetrically dividing embryonic neuroepithelial cells (NECs) of the optic placode expand in number before undergoing a fate switch to asymmetrically dividing embryonic neuroblasts (EONs), which generate ganglion mother cells that will produce neurons and glia of the optic lobe. (A”) during the larval neurogenesis period, additional optic lobe and lamina neuronal cells are generated in a similar fashion, with symmetrically dividing NECs undergoing a period of expansion before undergoing a cell fate switch into either asymmetrically dividing neuroblasts or lamina precursor cells (LPCs). Neuroblasts generate GMCs that produce neurons and glia, while LPCs generate lamina neurons directly. (B) in humans, cortex neurogenesis occurs between 10 and 25 gestational weeks (wks), followed by the completion of fetal development and birth with limited neurogenesis occurring in the postnatal stages. (B’) neurogenesis in humans also begins with an initial expansion of the NEC pool via symmetric division, which undergoes a similar cell fate switch into asymmetrically dividing radial glial cells (RGCs), analogous to fly neuroblasts. RGCs will generate either a neuron directly or an intermediate neural progenitor (INP), which are analogous to fly GMCs and will generate additional neurons. For an in-depth description of additional neurogenesis modes and neuroblast types in *drosophila*, see [56].

The brain, despite its complex architecture and function, consists of two post-mitotic cell types: neuronal (neurons) and non-neuronal (glia). These two cell types work together to ensure proper functioning of the nervous system. The total number of these cells in a brain will vary depending on the species and the complexity. For example, *Drosophila* has approximately 130,000 neuronal cells as opposed to humans, which consist of around 86 billion neurons [54,55]. This makes the human brain much more complex and significantly larger than the fly brain. However, the mode by which these neuronal and non-neuronal cells are generated, termed neurogenesis, is highly conserved. In other words, flies make brain cells in much the same way that humans do (Figure 2). For both organisms, the cells that comprise the adult brain are primarily generated during specific neurogenesis windows during early development: the embryonic and larval stages in *Drosophila*, and in the embryonic and postnatal stages in humans. This occurs from a small pool of neural stem cells called neuroepithelial cells (NECs) [56–59].

In mammals, these NECs originate from the lining of the neural tube, which in turn arises from the neuroectoderm. The anterior end of the neural tube undergoes an expansion and differentiation into the primary brain vesicles, such as the forebrain (prosencephalon), from where two telencephalic vesicles develop. The dorsal portion of these vesicles then undergoes specification to form the primordium of the cerebral cortex. During this time, the cortical primordium is comprised of a single layer of NECs. These highly polarized neural stem cells radially extend two thin processes away from the cell body, one of which contacts the apical surface of the neuroepithelium (apical process) and the other connects to the basal lamina (basal process). The apical process attaches adjacent NECs at the level of the apical surface via adherent and tight junctions [60,61].

NECs undergo repeated rounds of symmetric division to increase the progenitor pool and support the development of the neural tube. During each cell cycle, their nucleus moves along the entire apico-basal axis in a highly stereotyped fashion, called Interkinetic Nuclear Migration (INM). Mitosis occurs at these apical processes. During G1, the nucleus moves towards the basal lamina, where it remains during S-phase, and at G2, it moves back to the apical side, where it undergoes mitosis again. This cyclic motion of the NEC nuclei occurs completely asynchronously, resulting in a pseudostratified neuroepithelium with nuclei arranged at different positions along the apico-basal axis [3].

At the onset of neurogenesis, NECs lose tight junctions while retaining apical adherent junctions and transform into a population of neural stem cells known as apical radial glial cells (aRGCs). These highly polarized aRGCs possess a cell body situated within the Ventricular Zone (VZ), the primary germinal layer of the cortical primordium, which lines the ventricles. Their elongated processes exhibit apico-basal polarity, with a short apical process contacting the ventricular lumen (apical surface) and a long basal process extending radially towards the pial surface, forming a scaffold for neuronal migration. aRGCs also undergo INM, but become restricted to the apical domain, next to the ventricular surface. These aRGCs facilitate two major processes: neurogenesis (production of neurons and glia) and neuronal migration, where they serve as guidance cables aiding the migration of newborn neurons. In contrast to NECs, aRGCs undergo asymmetric cell division whereby an aRGC generates another aRGC and an intermediate progenitor cell (IPC) or immature neuron. IPCs migrate from the apical surface towards the SubVentricular Zone (SVZ), which is located at the basal border of the VZ. Here, IPCs undergo symmetric division, typically producing two immature neurons, or sometimes expanding their population by generating two IPCs. Immature neurons coordinate with radial fibres to migrate into the cortical plate and transform into mature neurons. Additionally, aRGCs serve as progenitors for glial cells. They can give rise to oligodendrocytes and ependymal cells, and a significant population of aRGCs ultimately differentiates into astrocytes, particularly after the peak of neurogenesis (for an in-depth review of mammalian neurogenesis, see [3,60–64]).

## Neurogenesis modes in *Drosophila*

In *Drosophila*, the central nervous system (CNS) develops from paired neural plates, located on the ventral region of the embryo. The brain develops from the anterior portion of the embryo, which is called the procephalic neuroectoderm. Similar to mammals, the neuroectoderm of the *Drosophila* embryo consists of a single layer of neuroepithelial cells (NECs). At the onset of neurogenesis, neuroblasts (NBs), another group of neural stem cells, delaminate from the neuroepithelium and rapidly undergo self-renewing asymmetric divisions to either give rise to another NB or transform into a smaller ganglion mother cell (GMC), which undergoes one final round of division to give rise to differentiated populations of neurons and glia. At the end of the embryonic neurogenesis window, NBs in the head and the thorax enter into a G0-like quiescent state until the early larval stages, where they reactivate and continue to generate neurons and glia during the second neurogenesis window in response to nutritional inputs. About 10% of the neurons found in the adult CNS are generated during the embryonic stage, and 90% generated during the larval neurogenesis stage [65–67].

Four types of neuroblasts can be found in the *Drosophila* CNS: Type 0 NBs can self-renew and directly differentiate into a daughter neuron. Type I NBs undergo multiple self-renewing asymmetric divisions to produce GMCs. Each GMC then undergoes one round of terminal division to produce two differentiated cells, either two neurons or a neuron and a glia. Type II NBs can also divide asymmetrically but produce an intermediate neural progenitor (INP) cell instead of a GMC. INPs go through another four to eight rounds of additional asymmetric division, leading to the formation of a GMC at each division, which can then differentiate into neurons and glia. Due to the generation of these transit-amplifying INPs, type II NBs are capable of generating much larger neuronal lineages than type I neuroblasts and are analogous to the neurogenesis mode utilized by the aRGCs in the outer SVZ of the mammalian cerebral cortex [68–70]. Type III neuroblasts, also called optic lobe NBs, first divide asymmetrically to generate distinct NB subtypes and then undergo both symmetric and asymmetric divisions to generate terminally differentiated neurons and glia. These different groups of NBs are distinguished primarily by the cell-polarity proteins and transcription factors they express. Deadpan (dpn) and worniu (wor) are expressed in both Type I and Type II neuroblasts. However, Type I and Type II NBs also express markers specific to their own lineages. For example, Type I NBs express prospero (pros) and asense (ase), which is not initially expressed by Type II NBs, but when they transform into INPs, they become Ase+. Similarly, Type III NBs uniquely express atonal (ato) [71].

## The *Drosophila* optic lobe, a model system for human MCPH studies

The adult fruit fly brain can be roughly divided into two major regions: a pair of optic lobes and the central brain. These two distinct regions are densely populated with neurons, which primarily arise from the different types of neuroblasts that make up the larval CNS described in the previous section [72]. The *Drosophila* optic lobes are an attractive model for the study of brain growth and development mechanisms that may be relevant to humans, considering that the neurogenesis modes are remarkably similar. Both utilize a pool of symmetrically dividing neuroepithelial cells (NECs) that later switch to asymmetric division modes as neuroblasts (NBs) that are neurogenic. This balance is thought to be critical in both humans and flies for generating the correct number of cells, therefore a brain of the correct size [73–76].

Moreover, the *Drosophila* optic lobes are highly neurogenic, generating ~67% of the total neurons of the fly brain (~120,000 neurons belonging to nearly 200 different morphological classes) [77–80]. Neurons within the fly optic lobe have also been observed undergoing migration to reach their final destination, similar to the neuronal migration that is prominent in mammalian cerebral cortex development [81–83]. Also, the optic lobes share structural similarities with the mammalian brain, such as layered architecture and columnar units, thus making it an excellent model to study molecular mechanisms underlying brain growth and development [84,85]. This is supported by previous studies in *Drosophila* brains mutated for *abnormal spindle (asp)*, the most commonly mutated gene in human MCPH, that showed the optic lobes were the most severely affected brain region with a significant reduction in volume as well as severe morphological defects [41,45].

The fly optic lobe originates from cells present in the embryonic neuroectoderm, termed the optic placode. A pool of Neuroepithelial cells (NECs) in this region actively divide to produce embryonic neuroblasts (EONs), which are marked by dpn expression. These EONs generate neurons and glia during the embryonic neurogenesis phase, then undergo a G0-quiescent phase and persist until the larval stages, where they reactivate and generate larval neurons of the optic lobe [73].

The adult optic lobe harbours four distinct groups of ganglia, which are called the lamina, medulla, lobula, and lobula plate, whose primary neurogenesis programs occur during the larval stages [79,82,84]. Upon larval hatching, the optic lobe primordium gradually segregates into two distinct neuroepithelial structures: the Outer Proliferation Center (OPC) and the Inner Proliferation Center (IPC). The OPC is involved in the generation of outer medulla and lamina neurons, whereas the IPC is involved in the generation of inner medulla, lobula, and lobula plate neurons. During the early stages of larval neurogenesis, NECs of the OPC go through repeated rounds of symmetric cell divisions to increase the progenitor population. Three distinct neurogenesis modes have been uncovered in these neuroepithelial populations. First, the lateralmost edge of the OPC gives rise to a unique population of neural progenitor cells known as the lamina precursor cells (LPCs), which share characteristics with GMCs in that they can directly differentiate into lamina neurons. This occurs via both intrinsic (e.g. transcription factor) and extrinsic factors (cell signalling molecules such as Hedgehog (Hh) and the epidermal growth factor (EGF)-like ligand Spitz) [86–88].

Second, towards the early third instar larval stage, the medial-most tips of the OPC undergo a fate switch without division to transform into medulla NBs (mNBs), which then undergo repeated rounds of self-renewing asymmetric divisions leading to the formation of GMCs, which can then terminally divide into differentiated populations of neurons and glia [79,89,90]. The transformation of medial NECs into mNBs in this region is mediated by a wave of differentiation called the ‘proneural wave’, which progresses from the lateral to the medial side of the fly brain, creating a region known as the transition zone (TZ). This transition zone is characterized by the temporary expression of the proneural transcription factor Lethal of scute (L’sc) in NECs, which promotes differentiation into mNBs. Many cell signalling pathways regulate the progression of this wave, including Notch and EGFR, where the coordinated sequential action of these pathways trigger the differentiation of NECs into mNBs, whose identities are further refined and diversified by the sequential expression of spatial and temporal transcription factors (sTFs and tTFs) [91–97].

Third, the inner proliferation centre (IPC) generates three distinct neuronal populations: distal cells (C2, C3, T2, and T3 neurons); lobula plate neurons (T4 and T5 neurons); and lobula neurons. This diversity is mediated by distinct NEC subdomains within the IPC. NECs of the proximal IPC (p-IPC) undergo an epithelial-to-mesenchymal transition (EMT) in response to Wingless signalling inputs and migrate via cell streams to a second horseshoe-shaped domain located near the OPC, known as the distal IPC (d-IPC). These cells then transform into neuroblasts within the dIPC, where they divide asymmetrically to produce distal cells and lobula plate T4/T5 neurons depending on transcription factor inputs. Lobula neurons are generated from the surface-IPC (s-IPC), located near the pIPC [85,98]. While the specific mechanisms regulating neurogenesis from the IPC are still under investigation, the involvement of conserved cell signalling pathways and transcription factor inputs, cell migration, and timely cell fate conversions between symmetrically and asymmetrically dividing neural stem cell populations make the IPC an intriguing model for future MCPH studies in the fly.

Next, we briefly discuss the neuroanatomy of each of the four adult ganglia of the optic lobe that form as a result of these neurogenesis modes in the larval optic lobe and describe their similarities to the mammalian cortex.

## Lamina

The lamina is the first optic lobe ganglion situated medially to the retina. The lamina is arranged into an array of about 750 lamina cartridges that receive inputs from the outer R1-R6 photoreceptor axons, collecting visual information. The two other photoreceptor inner axons, R7 and R8, project directly to specific medulla layers, passing through the lamina without synapsing there. In addition to these PR axons, the lamina also consists of 12 other neuronal subtypes. These neuronal subtypes comprise five lamina output neurons, six putative feedback neurons, and one lamina intrinsic neuron. L1–L5 lamina neuron subtypes send axonal connections into the medulla. L1, L2, and L3 neurons receive direct input from R1–R6 photoreceptors, while L4 and L5 receive inputs from the R1-R6 pathway, primarily through other lamina neurons. Lamina neuron types can be categorized by the transcription factors they express. L1, L2, and L3 neurons express Svp, Bab2, and Erm, respectively. L4 and L5 neuronal types express Bsh/Ap and Bsh/Pdm3, respectively [99].

Additionally, T1, C2, and C3, the three putative feedback neurons, connect to the lamina through the medulla. Another class called the lamina wide-field neurons (Lawf 1 and 2), which function in wide-field feedback from the medulla to the lamina, are derived from the glial precursor cell (GPC) regions of the OPC and undergo a significant migration to reach their final destination, similar to mammalian neurons. Finally, the medulla connects to the lamina through the outer optic chiasm (OOC), consisting of lamina neuron axons that cross over each other in a defined pattern to establish the retinotopic map. To summarize, the structural organization and function of the lamina is relevant for understanding how visual information is processed and how spatial maps are established, both of which are critical for higher-level visual processing in both flies and mammals [82,100].

## Medulla

The second optic lobe ganglion of the optic lobe, the medulla, is the largest processing centre of visual information, containing over 200 neuronal cell types that contribute to the formation of a complex neural circuit with 800 vertical columns and ten horizontal layers. Additionally, the *Drosophila* medulla shares structural and developmental similarities with the mammalian cerebral cortex, including the presence of both layered and columnar organization, utilizing similar mechanisms of neuronal migration and specification as well as having conserved cell interaction patterns [101]. The distal medulla includes the M1–M6 layers, consisting of lamina neurons and inner PRs. The proximal medulla, including M7–M10, is separated from the distal medulla by the serpentine layer (M7). These layers are distinguished by different Cadherin-N (CadN) or Bruchpilot (Brp) staining levels, in combination with layer-specific markers, including Chaoptin, which labels PR axons. Notch-dependent binary fate choices and temporal patterning of medulla NBs are responsible for generating the vast diversity of medulla neurons [85,102,103].

## The lobula complex

The medial-most ganglia of the *Drosophila* optic lobe is called the lobula complex, which consists of the lobula and lobula plate neuropiles. The lobula is comprised of six layers (Lo1- Lo6) and connects to the lobula plate via the inner optic chiasm (IOC). The lobula shares certain fundamental similarities with the mammalian cortex, including its retinotopic architecture, where it maintains a spatial organization of visual information [104]. Additionally, the lobula is involved in processing motion, object features, and detecting visual looms just like the mammalian brain [105]. Lastly, the lobula connects visual detection with behavioural control, which is also a feature of mammalian cortex [97,106].

The lobula plate neuropil can be divided into four layers containing dendrites that can sense motion along one of the four cardinal directions. T4, T5, and Tlp neurons of the lobula plate connect with either the medulla or lobula as part of the visual motion processing circuit. The lobula plate also houses giant tangential neurons called the Lobula Plate Tangential Cells (LPTCs), which play crucial roles in the processing of motion vision, particularly in the detection of movement within the visual field. They are involved in motion detection and the integration of visual information to guide the fly’s behaviour. These neurons receive input from the deeper layers of the optic lobe, especially from the medulla and lobula, and transmit processed motion information to the CNS for further integration and behavioural response [79].

LPTCs primarily acquire their directional sensitivity from integrating local motion information from presynaptic T4 and T5 neurons to derive global motion information. T4 neurons convey ON local motion to signify the direction of motion of bright edges, whereas T5 neurons provide OFF local motion to represent dark edges. There are four different subtypes of T4 and T5 neurons, each accounting for one cardinal direction. The major types of LPTCs in *Drosophila* are as follows: Horizontal Motion Detectors or HS neurons and Vertical Motion Detectors or VS neurons [79,85,107]. The lobula plate serves as a higher-order visual processing centre, similar to the dorsal stream in the mammalian cortex. The lobula plate’s structure and function is essential for delineating how brains compute and use motion information for behaviour [108].

## Human MCPH genes and their Drosophila orthologs

MCPH has been linked to mutations in numerous genes. As of 2025, 30 MCPH genes have been characterized and ordered numerically as MCPH 1-30 (Table 1). Generally, MCPH mutations are loss-of-function resulting from nonsense, frameshift, deletions, or splice site-mutations, all of which result in non-functional proteins. The different genes associated with MCPH perform a variety of essential cellular processes, whose most well-characterized cellular roles include de novo centriole assembly, microtubule turnover, damage to the DNA, and interaction with various cell signalling pathways [109]. Many of these MCPH genes encode for centrosome or spindle-pole associated proteins. MCPH has been hypothesized to be caused by an abnormal neurogenesis programme, resulting from various factors, including increased neuronal death, an aberrant ratio of progenitor to differentiating cells, and abnormal timing in cell division and differentiation. This can lead to a reduction in final brain cell number and a corresponding reduction in brain size [4,26,27].

**Table 1.** List of human genes given an official MCPH designation in OMIM and the corresponding ortholog from *Drosophila melanogaster.*

Human MCPH Gene	MCPH Designation	OMIM Number	Fly Ortholog
*Microcephalin 1*	MCPH1	607117	*Microcephalin*
*WD Repeat-Containing Protein 62 (WDR62)*	MCPH2	604317	*Wdr62*
*CDK5 Regulatory Subunit-Associated Protein 2* (*CDK5RAP2*)	MCPH3	604804	*centrosomin (Cnn)*
*Kinetochore Scaffold 1 (KNL1)/CASC5*	MCPH4	604321	*KNL1*
*Abnormal Spindle-Like, Microcephaly- Associated (ASPM)*	MCPH5	608716	*abnormal spindle (asp)*
*Centromeric Protein J (CENPJ)*	MCPH6	608393	*Spindle assembly abnormal 4 (Sas-4)*
*SCL/TAL1-Interrupting Locus (STIL)*	MCPH7	612703	*anastral spindle 2 (ana2)*
*Centrosomal Protein, 135-KD (CEP135)*	MCPH8	614673	*Centrosomal protein 135kDa (Cep)*
*Centrosomal Protein, 152-KD (CEP152)*	MCPH9	614852	*asterless (asl)*
*Zinc Finger Protein 335 (ZNF335)*	MCPH10	615095	*CG10366*
*Polyhomeotic Homolog 1 (PHC1)*	MCPH11	615414	*Polyhomeotic proximal & distal (ph-p & ph-d)*
*Cyclin Dependent Kinase 6 (CDK6)*	MCPH12	616080	*Cyclin-dependent kinase 4 (CDK4/6)*
*Centromeric Protein E (CENPE)*	MCPH13	616051	*CENP-ana (cana)/CENP-meta (cmet)*
*SAS6 Centriolar Assembly Protein (SASS6)*	MCPH14	616402	*Spindle assembly abnormal 6 (Sas-6)*
*Major Facilitator Superfamily Domain-Containing Protein 2A (MFSD2A)*	MCPH15	616486	
*Ankyrin Repeat- and LEM Domain- Containing Protein 2 (ANKLE2)*	MCPH16	616681	*Ankle2*
*Citron Rho-Interacting Serine/Threonine Kinase (CIT)*	MCPH17	617090	*Sticky (sti)*
*WD Repeat- and FYVE Domain- Containing Protein 3 (WDFY3)*	MCPH18	617520	*Blue cheese (bchs)*
*Coatamer Protein Complex, Subunit Beta- 2 (COPB2)*	MCPH19	617800	*Coat Protein (coatomer) β’(β’COP)*
*Kinesin Family Member 14 (KIF14)*	MCPH20	617914	*Nebbish (neb)*
*Non-SMC Condensin I Complex Subunit D2 (NCAPD2)*	MCPH21	617983	*CAP-D2 condensin subunit (Cap-D2)*
*Non-SMC Condensin II Complex Subunit D3 (NCAPD3)*	MCPH22	617984	*Chromosome associated protein D3 (Cap-D3)*
*Non-SMC Condensin I Complex Subunit H (NCAPH)*	MCPH23	617985	*Barren (barr)*
*Nucleoporin, 37-KD (NUP37)*	MCPH24	618179	*Nucleoporin 37kD (Nup37)*
*Trafficking Protein Particle Complex, Subunit 14 (TRAPPC14)/MAP11*	MCPH25	618351	
*Lamin B1 (LMNB1)*	MCPH26	619179	*Lamin (Lam)*
*Lamin B2 (LMNB2)*	MCPH27	619180	*Lamin (Lam)*
*Ribosomal RNA Processing 7 Homolog A (RRP7A)*	MCPH28	619453	*CG9107*
*Programmed Cell Death 6-Interacting Protein (PDCD6IP)*	MCPH29	620047	*ALG-2 interacting protein X (ALiX)*
*BUB1 Miotic Checkpoint Serine/Threonine Kinase (BUB1)*	MCPH30	620183	*Bub 1-related kinase (BubR1)*

However, the precise mechanism by which mutations in these genes disrupt neurogenesis and, in turn, reduce overall brain size, have proven elusive. While correlation based on the most ‘well-known’ function of the MCPH gene in question provides a logical framework, in many cases these hypotheses have failed when put to the test in model organisms [9–12]. Furthermore, a common theme of these studies is that the less well-known functions of the MCPH genes under investigation are likely to be responsible for the small brain phenotype. This suggests that protein multifunctionality may be a significant contributor to the aetiology of MCPH, and perhaps other human diseases caused by single-gene mutations [14]. Below, we summarize recent results from the most well-studied MCPH orthologs from *Drosophila* and highlight the moonlighting functions uncovered to date.

### Abnormal spindle-like, microcephaly associated (ASPM)/MCPH5

*Abnormal spindle-like, microcephaly associated (ASPM)*, also known as MCPH5 (OMIM #608716), is the most commonly mutated gene found in human MCPH patients, accounting for more than 40% of the patient population. Human *ASPM* possesses 28 exons and at least two alternative splicing isoforms: isoform 1 (full-length, amino acids 1–3,477) and isoform 2 (lacking the largest exon, exon 18, which encodes amino acids 1,356–2,940). Human ASPM protein consists of four domains: an ASH (ASPM-SPD2-Hydin) domain at the N-terminus (NT), two calponin homology domains (CH), an isoleucine and glutamine domain (IQ motif), and a species-conserved C-terminal domain that contains HEAT/Armadillo-like repeats. An intrinsically unstructured region is located between the ASH and CH domains. The ASH domain of ASPM is thought to play a crucial role in spindle integrity and function. Its function is linked to binding microtubules and facilitating spindle organization at the poles. The CH domains, commonly found in actin-binding proteins, also facilitate a stronger interaction with microtubules than the ASH domain. The 81 IQ repeats are implicated in calmodulin binding [110]. The function of the C-terminal HEAT/Armadillo-like repeat is not known [111]. The diverse number of protein domains in ASPM hints at potential multifunctional roles for this protein.

The *Drosophila* ortholog of *ASPM*, known as *abnormal spindle* (*asp*), is a 220-KD protein consisting of 1,954 amino acid residues with a pI of 10.8, which makes it extremely basic and challenging to work with biochemically. Only a single isoform is predicted in FlyBase, although this has not been extensively studied and there may be a second isoform, like mammals. Asp was first identified in the 1980s, well before human ASPM, and named for the obvious mitotic phenotype observed: unfocused mitotic spindle poles and centrosome-pole detachment [112]. Other mitosis phenotypes have been reported for different mutant *asp* alleles, including defects in chromosome segregation, cytokinesis, and mitotic timing [113–117]. Later work showed that Asp promotes proper mitotic spindle organization through its interaction with Calmodulin via Asp’s IQ domains in the C-terminus and its ability to crosslink microtubule minus ends via the CH and ASH domains, thereby ensuring proper mitotic spindle architecture [9,118].

These mitotic defects observed in *Drosophila asp* mutants formed the preliminary hypothesis for how loss of human ASPM function causes MCPH [119]. However, later studies in *Drosophila* showed that loss of mitotic spindle morphology *per se* is not the primary driver of the MCPH phenotype, and that the N-terminus of Asp, including the ASH domain and neighbouring unstructured region (known as the ‘minimal fragment’), is both necessary and sufficient to restore brain size [9,41,45]. These studies were the first to suggest that Asp must therefore have additional cellular functions outside of mitotic spindle organization that are essential for proper brain growth and development.

But what might these other multifunctional roles for Asp be? Other studies in flies have shown that Asp interacts with components of the acto-myosin cytoskeleton to ensure proper neuroepithelial tissue architecture, which may impact the symmetrically dividing NEC pool and it’s timely transition into asymmetrically dividing medulla Neuroblasts (mNBs) [120]. Indeed, other studies have also shown an essential and specific role for Asp and ASPM in symmetrically dividing precursor cells in both *Drosophila* and mice, supporting a model where Asp/ASPM function in NECs is key to making a properly sized brain [46,75]. While the specific Asp/ASPM function(s) in NECs that ensure proper brain size remains to be elucidated, support for additional novel multifunctional roles include modulation of cell cycle timing via Cdk2/Cyclin E and regulation of essential cell signalling pathways known to control brain development, such as Wnt, Hedgehog, and Notch [12,121–127].

Recent transcriptional profiling of *asp* mutant and rescue strains from our laboratory provide further support for Asp as a potential regulator of cell signalling pathways and their downstream transcriptional targets that specify neural fate in the optic lobe [94]. We identified a strong enrichment for neurogenesis, Notch signalling, and retina development and morphogenesis, which were downregulated in *asp* mutants. The neurogenesis node included major regulators of the Notch pathway, including *Notch* (*N*) receptor, and the target genes of the Notch pathway belonging to the *enhancer of split* (*E(spl)*) complex (e.g. *E(spl)mβ-HLH* and *E(spl)m4-BFM*). Many well-known spatial and temporal transcription factors that generate the vast diversity of neuron types of the optic lobe, including *Optix*, *eyeless* (*ey*), *twin of eyeless (toy), Dichaete* (*D*), *runt* (*run*), *tailless* (*tll*), *sloppy paired1* (*slp1*), and *odd paired* (*opa*) were also strongly enriched (downregulated) in the analysis [41,95,96].

Together, these studies point towards additional multifunctional roles for Asp in brain growth and development. Future studies in flies will be key for identifying these roles, which we believe should focus on elucidating the mechanism of Asp’s role as a neurogenesis promoting factor, particularly during interphase of the cell cycle. This could be achieved by identifying novel protein interacting partners of the N-terminal ‘minimal fragment’ region and performing functional genetic and biochemical assays in larval brains to dissect the relevant contributions of these multifunctional interactions to the MCPH phenotype. We also suspect that these studies will further clarify the relationship between Asp’s role as both a brain growth promoting factor and a regulator of brain morphology, which will further highlight the multifunctional roles of Asp in the developing brain [41]. We also believe that these findings in the fly will be relevant to humans, considering that ‘humanized’ *asp* mutant flies expressing a human ASPM fragment show relatively normal brain size, suggested conserved mechanisms of function(s) between human ASPM and fly Asp [46].

### Microcephalin/MCPH1

*Microcephalin* or *MCPH1* (OMIM #251200) was the first gene mutation that was identified in association with MCPH [1,128]. Decades of study in flies and mammals have revealed *MCPH1* to also be a multifunctional protein, with roles described in DNA damage repair, chromosome architecture, cell cycle regulation, & numerous mitotic defects including chromosome condensation, alignment, and segregation [129–134]. The human protein is 835 amino acids, and like many proteins involved with DNA repair and cell cycle progression, contains three BRCA1 C-terminal (BRCT) domains. This domain consists of a phosphoprotein binding module that allows for heterotypic interactions with other phosphorylated proteins and also the DNA backbone. They can also facilitate protein-protein interactions between other BRCA1-containing proteins, forming large multimer complexes that are consistent with the nature of a multifunctional protein [135–138].

However, the contributions of the three BRCT domains in MCPH1 to the microcephaly phenotype are not equivalent. The BRCT domain at the N-terminus appears to be the most important, with an enrichment of human patient mutations located in this region [139]. Furthermore, mice lacking only the C-terminal BRCT domain have normal brain size, whereas mice lacking only the N-terminal BRCT domain have microcephaly [140,141]. These N-terminal BRCT-deficient mice also showed premature chromosome condensation and a defective DNA damage response, suggesting that one or both of these cellular roles for MCPH1 might be essential for proper brain growth and development [141].

In *Drosophila*, the microcephalin gene consists of nine exons and encodes four different protein isoforms ranging from 779 to 1028 amino acids. While fly MCPH1 shares only ~18% amino acid identity with the human counterpart, it includes the three BRCT domains, including the one located at the N-terminus. The 826 amino acid isoform (MCPH1-PB) has been shown to be crucial for the early syncytial nuclear divisions to coordinate centrosome and nuclear division cycles, mitotic progression, centrosome/centriole function, and centrosome-pole cohesion, with *mcph1* mutants showing increased genomic instability and mitotic arrest that is Chk2 dependent, likely due to substantial DNA damage resulting from premature chromosome condensation, a phenotype conserved in mammals [129,132,140,142]. However, deletion mutants presumably lacking the N-terminal BRCA1 domain do not show an obvious microcephaly phenotype in flies, although morphological defects in the adult central brain, particularly in the mushroom bodies, which contribute to olfactory memory and other complex adaptive behaviours, were observed [129,142].

Much like the other MCPH genes covered in this review, a definitive cellular mechanism linking *MCPH1* to the small brain phenotype remains unresolved, largely due to the multifunctional nature of the protein. Nonetheless, previous studies in mice have suggested that a disruption to the ratio of symmetrically dividing NECs to asymmetrically dividing neural stem cells is ultimately responsible for the microcephaly phenotype of *MCPH1* mutants, similar to the explanation proposed for *ASPM* microcephaly [75,143]. More work will be required to establish the mechanism, and we propose that flies could be a useful system for these studies. It is worth noting that MCPH1 interacts with the Condensin II complex, which has numerous microcephaly cases reported in humans and mice when mutated, with *NCAPD3* having an official MCPH designation (MCPH22, see Table 1) [144–151]. A recent study in *Drosophila* has also shown that mutations in Condensin II subunits cause microcephaly via retrotransposon activation and subsequent cell death in neural stem cells [152]. It would be of interest to explore the link between MCPH1, Condensin II subunits, and retrotransposable element activation in *Drosophila*, especially with modern genetic and imaging tools (CRISPR, transgenic rescue experiments, and microcomputed tomography), which were lacking in earlier *MCPH1* fly studies. It is possible that such a connection could also explain the microcephaly phenotype for *MCPH1* in mammals as well, including humans.

### WD Repeat-Containing protein 62 (WDR62)/MCPH2

The second most commonly mutated gene in primary microcephaly is *WD Repeat-Containing Protein 62 (WDR62)* or *MCPH2* [153]. In addition to microcephaly, human patients carrying mutations in *WDR62* can also show other brain malformations, such as lissencephaly [154]. This is perhaps not surprising given that the human genome encodes at least twelve different protein isoforms. The presence of the WD40 repeat domain also hints at a multifunctional role for this protein, since these domains are known to coordinate protein-protein interactions by serving as a scaffold for multiprotein complexes [155]. Indeed, WDR62 has been shown to regulate several biological processes in flies and mammals, including mitotic spindle assembly and orientation, centrioles, centrosomes, and cilia [10,156–161]. The *Drosophila* ortholog of WDR62 also encodes for several isoforms, the longest of which is a 2,397 amino acid protein that shares ~35% amino acid identity with its human counterpart, including the presence of three WD40 domains. Flies carrying *wdr62* mutations also show the microcephaly phenotype, with a ~ 40% reduction in central brain volume [158].

Early studies in mice suggested that mutations in *WDR62* led to a reduction in the cortical neural stem cell pool, resulting in the production of fewer neurons and the microcephaly phenotype [44]. A similar neurogenesis defect was also detected in the hippocampus of WDR62-deficient mice [162]. This cellular phenotype was later verified in human brain organoids derived from patients carrying pathogenic mutations in *WDR62*^163^. However, the underlying cause of this phenotype has yet to be clarified. Earlier studies focused on WDR62’s role in mitosis, where its loss was shown to disrupt mitotic spindle architecture, spindle assembly checkpoint (SAC) activation, and eventual cell death due to a failure to progress through mitosis in a timely manner. This was suggested to be due to WDR62’s interaction with Aurora A, a kinase essential for spindle assembly and chromosome segregation [44].

In subsequent years, as more centriole genes were given an MCPH designation, the field shifted to WDR62’s role at the centriole in an attempt to provide a unifying cellular hypothesis for how WDR62 mutations cause microcephaly. Explanations included WDR62’s role at centriolar satellites, centriole duplication, cilia disassembly, and the proper formation of the apical complex to ensure neural stem cell fate, thus leading to the suggestion that MCPH is a ‘centriolopathy’ [10,157,163,164]. Studies in *Drosophila* echoed these mammalian findings, which showed WDR62 plays a crucial role in maintaining centrosome asymmetry in fly neuroblasts [158].

However, *Drosophila* studies in recent years have added an additional layer of complexity, while highlighting the multifunctional nature of *WDR62* in a cell-type specific manner. While mammalian studies focused exclusively on neurogenic lineages (neural stem cells and their descendants), Lim et.al took advantage of the Gal4/UAS system in *Drosophila* to test whether WDR62’s function in neuroblasts (neural stem cells) or other cell types, such as neurons and glia, was responsible for the microcephaly phenotype [165]. Surprisingly, RNAi depletion of WDR62 specifically in glia caused a microcephaly phenotype, whereas depletion in neuroblasts had no effect on brain size despite a decrease in the number of neuroblasts. In parallels to mammals, WDR62’s function in *Drosophila* glia required Aurora A kinase [11,44]. Later studies showed that WDR62 mediates a signalling axis to promote cell growth and proliferation to ensure proper brain size, which included activation of Aurora A and AKT signalling to drive MYC expression [166]. Other mammalian studies have also shown that WDR62 modulates JNK signalling by acting as a protein scaffold, which may ensure proper neurogenesis and cortical development [167,168].

Together, these studies highlight the multifunctional nature of WDR62, which has limited our ability to pinpoint the exact cellular mechanism by which loss of this protein contributes to the microcephaly phenotype. However, WDR62’s ability to modulate neurogenic signalling pathways, similar to ASPM, may provide an intriguing path for future studies hoping to identify a unifying mechanism of brain growth and development in MCPH. It also warrants further investigation into the role of mammalian glia biology in the context of MCPH, which has so far remained largely focused on neural stem cells in the aetiology of the disorder.

### Ankyrin repeat-and LEM domain-containing protein 2 (ANKLE2)/MCPH16

Of the genes discussed so far in this review, *Ankryin Repeat-and LEM Domain- Containing Protein 2 (ANKLE2)/MCPH16* is the newest member of the MPCH family. It was first reported in 2014 by Bellen and colleagues in a study that linked a handful of rare human allele variants of undiagnosed Mendelian diseases to their corresponding *Drosophila* orthologs. A patient displaying microcephaly and additional brain malformations was confirmed to have compound heterozygous mutations in *ANKLE2*. To determine if *ANKLE2* was responsible for the human microcephaly phenotype, the *Drosophila* ortholog (*dAnkle2*) was mutated. Larval brain size was significantly reduced in mutants compared to controls. Importantly, this reduction in brain volume was significantly restored when the human ortholog of Ankle2 was ubiquitously expressed in mutant flies, suggesting that human and fly ANKLE2 share a conserved mechanism(s) that promotes proper brain growth and development while establishing *Drosophila* as a useful model system for ANKLE2-related MCPH in humans [34]. Additional human mutations in *ANKLE2* have since been reported in MCPH patients, establishing its role as a brain growth regulator [169,170].

Early *dAnkle2* studies explored its potential roles in mitosis, centriole/centrosome function, and apoptosis in neural stem cells as an initial description of why brain volume might be smaller in *dAnkle2* mutants. Flies carrying *dAnkle2* mutations were shown to have a significant reduction in the total number of neuroblasts, reduced cell proliferation, and a massive amount of apoptosis in the developing brain, which could be rescued by expression of a human *ANKLE2* cDNA. However, spindle orientation was not significantly affected in those cells that were able to divide, and no obvious defects were seen in centriole/centrosome function [34].

If mitotic spindle and centriolar contributions cannot fully explain the *ANKLE2* microcephaly phenotype, what other functions does it possess in cells? Like the other MCPH genes in this review, ANKLE2 has multifunctional roles and probably plays a role in a number of different cellular processes that can converge on reduced cell proliferation rates and death. It might also influence broad processes such as transcription, translation, or phosphorylation levels in the cell. To do so would imply the existence of multiple protein domains. Indeed, ANLKE2 contains a LEM (LAP2, Emerin and MAN1) domain, which facilitates localization to the inner nuclear membrane (INM) and can also bind to nuclear lamins [171]. It also has multiple ankryin repeats, which can mediate protein-protein interactions, a caulimovirus domain that promotes interactions with protein phosphatase 2A (PP2A), along with uncharacterized C-terminal domains that are essential for the ability of ANKLE2 to promote proper brain size [34,47,172,173].

Much of the focus has revolved around ANKLE2’s function at the nuclear envelope (NE), where it acts to facilitate timely reassembly following mitotic exit. This occurs through ANKLE2’s ability to regulate the phosphorylation state of barrier to autointegration factor (BAF), which is essential for NE breakdown and reassembly dynamics during mitosis. ANKLE2 does this through its interaction with PP2A and the kinase VRK-1, where it promotes PP2A’s ability to dephosphorylate BAF upon mitotic exit and partially blocks VRK-1 kinase activity, allowing for proper coordination of NE reassembly dynamics following chromosome segregation [173]. The interaction between dAnkle2, PP2A, and VRK-1 (Ballchen) is also conserved in *Drosophila*, with evidence suggesting that dAnkle2 may be a bona fide regulatory subunit of PP2A [174]. Further support that an ANKLE2/VRK-1/PP2A axis at the NE might be required for proper brain growth comes from the fact that mutations in VRK-1 have also been found in human microcephaly patients [34,175], and PP2A was recently shown to interact with Asp (*ASPM/MCPH5*) in *Drosophila* [176].

However, studies in *Drosophila* have provided additional mechanisms by which dAnkle2, and potentially human ANKLE2, promote proper brain growth. dAnkle2 localizes to both the NE and endoplasmic reticulum (ER) in fly neuroblasts, and its loss causes disruption to these organelles, suggesting dANKLE2 is required for NE and ER morphology. Additionally, dAnkle2 mutations disrupt aPKC and other polarity complex proteins, affecting the ability of neuroblasts to consistently divide asymmetrically due to spindle alignment defects. This phenotype could be alleviated by dose-dependent reductions in VRK-1/Ballchen, and could also restore brain size. This suggests a model where proper segregation of asymmetric determinants is a critical function of dAnkle2 to ensure proper brain size. However, expression of a constitutively active aPKC on its own in *dAnkle2* mutants could not rescue the brain size phenotype, suggesting that polarity defects are not the primary contributor to *dAnkle2’s* microcephaly phenotype. These results highlight dAnkle2’s multifunctional nature, and suggest that its role in multiple cellular processes, even if minor, may collectively be responsible for the microcephaly phenotype [47]. Interestingly, expression of Zika virus protein NS4 behaves nearly identically to dAnkle2 loss, suggesting a common pathway by which environmental and genetic factors converge to affect brain growth and development [47,170,177].

## Future outlook for *Drosophila* as a model for MCPH

In this review, we explored how the fruit fly is relevant to studying the molecular basis of human MCPH and dissected the roles of some of the most commonly mutated genes in the pathogenesis of microcephaly related to neurogenesis. With recent advances in human organoid technology to directly model microcephaly and a shift in NIH initiatives to prioritize human-based research and reduce the use of animals in research, will *Drosophila’s* extensive track record in uncovering molecular mechanisms relevant to human disease soon be coming to an end [34,163,178–180]?

We certainly do not think so. While human organoid models can undoubtedly provide significant insights into the aetiology of human MCPH, particularly organoids derived from induced pluripotent stem cells (iPSCs) taken from MCPH patients who may have novel allele variants, they still lack a few key attributes that limit their use as the sole MCPH model system or for NDDs in general [181,182]. While these limitations have been reviewed elsewhere, it is worth highlighting that the remarkable complexity by which brain development and function cannot yet be fully recapitulated *in vitro*, and the issue of reproducibility due to the heterogeneous nature by which organoids develop – there can be significant differences in brain organoid size, even from those generated from wildtype cells [183,184]. Since MCPH is clinically defined as a reduction in overall head and brain size compared to the general population, it necessitates the use of an entire organism to accurately assess phenotypes upon genetic manipulation. It also requires an organism to provide the correct suite of cell and developmental signals to generate a fully functional and remarkably complex brain.

Thus, *Drosophila* will remain a powerful and indispensable model for uncovering molecular mechanisms relevant to human MCPH. With recent advances in genetic manipulation strategies (e.g. CRISPR/Cas systems), the establishment of highly accurate, 3D X-ray imaging technologies to precisely measure brain size in intact animals at high resolution and significant knowledge of brain development, architecture and connectivity built on decades of studies by thousands of *Drosophila* researchers that is unrivalled in higher vertebrate organisms makes flies an essential model for future studies aimed at elucidating the mechanisms of MCPH [35,36,45,185]. We hope that future research in flies will consider the suite of multifunctional roles that MCPH proteins have when interpreting experimental results and move beyond correlation. If so, we may be able to finally answer the question of whether there is a unifying molecular mechanism underlying MCPH, or if it’s a bit more complex, much like the brain itself.

## Data Availability

The data reported in this review are available upon request.
